# Molecular detection of *Trypanosoma minasense* in captive black-faced black spider monkeys *Ateles chamek* (mammalia; primate)

**DOI:** 10.3389/fvets.2024.1527193

**Published:** 2025-01-15

**Authors:** Louise Bach Kmetiuk, Ana Silvia Miranda Passerino, Marcelo Bonat, Vívien Midori Morikawa, Alice Pereira Berbigier, André Luiz Rodrigues Roque, Fabiano Borges Figueiredo, Alexander Welker Biondo

**Affiliations:** ^1^Graduate Program in Biosciences and Biotechnology, Instituto Carlos Chagas, Fundação Oswaldo Cruz, Curitiba, Paraná, Brazil; ^2^Curitiba City Secretary of Health, Curitiba, Paraná, Brazil; ^3^Curitiba City Secretary of Environment, Curitiba, Paraná, Brazil; ^4^Laboratory of Trypanosomatid Biology, Oswaldo Cruz Institute, Oswaldo Cruz Foundation, Rio de Janeiro, Rio de Janeiro, Brazil; ^5^Department of Veterinary Medicine, Federal University of Paraná, Curitiba, Paraná, Brazil

**Keywords:** captive primates, blood parasites, molecular analysis, non-human primates, *Trypanosoma* spp.

## Abstract

The present study aimed to assess *Trypanosoma* spp. infection in 17 captive black-faced black spider monkeys (*Ateles chamek*), living at the Zoological Garden of Curitiba, Paraná, Brazil. Blood samples (3 mL) were collected by femoral puncture by certified veterinarians. A total of 5 μL of extracted blood DNA was submitted to Nested-PCR 18S rDNA. In overall, 4/17 (23.5%) spider monkeys were positive to *Trypanosoma* spp. by PCR. Sequencing results for 18S rDNA revealed *Trypanosoma minasense* infection. This is the first report of *T. minasense* in black-faced black spider monkey worldwide and the first assessment of *Trypanosoma* species in NHP of southern Brazil.

## 1 Introduction

Non-human primates (NHPs) may be infected and participate in transmission cycle of zoonotic pathogens, as reported for rabies virus (1), *Leishmania* spp. (2), Yellow fever (3), Mayaro fever (4), and *Trypanosoma* spp. (5). Due to the phylogenetic proximity with humans and increased interaction with humans and vectors in periurban and urban areas (6), NHP infections by *Trypanosoma* spp. have been assessed in rural and periurban areas, along with wildlife centers and zoological gardens (6).

NHPs have been considered as sylvatic hosts of *Trypanosoma* spp. in South America, particularly of *Trypanosoma cruzi*, the protozoan agent of Chagas disease (5). *T. cruzi* infection has been reported in both free-ranging and captive primates of Brazil, including 17/26 (65.4%) primates from the Brasília Zoo, central-western region (7), 14/112 (12.5%) from Amapá, Pará, and Amazonas states, northern Brazil (8). A high diversity of natural trypanosome infection has been indicated in the Brazilian Amazon, with 22/46 (47.8%) NHPs infected by *T. cruzi*, *T. minasense*, *T. devei* and *T. rangeli* at the National Cent**er** of Primates, Pará State (5). In addition, 2/38 (5.3%) free-ranging and captive primates from the Mato Grosso State, central-western Brazil, were positive to *T. minasense* and *T. rangeli* (9). *T. minasense* has been considered one of the most common and highly specific parasites of NHP hosts, reportedly presenting low and persistent parasitemia (10). In addition, *T. minasense* has been identified as a primitive *Trypanosoma* species, with highly similar 18S rRNA to *T. grayi* and *T. avium*, respectively reptile and avian parasites (10).

*Trypasonoma* spp. infection in captive NHPs may contribute to infection and spreading in non-endemic areas, besides the potential zoonotic source for handling keepers, biologists and veterinarians (7). Accordingly, the present study aimed to assess and identify *Trypanosoma* spp. infection in a colony of captive, black-faced black spider monkeys (*Ateles chamek*) kept at the Zoological Garden of Curitiba, southern Brazil.

## 2 Method

### 2.1 Ethical statement

This study has been approved by the Ethics Committee of Animal Use (protocol number 071/2021) at the Federal University of Paraná and included as part of the surveillance activities of the Curitiba City Secretary of Environment.

### 2.2 Study area

The present study was conducted in the Zoological Garden of Curitiba (25° 33′ 38″ S, 49° 14′ 07″ W), Curitiba, Paraná State, southern Brazil, which kept at the time approximately 2,300 animals of 300 species, distributed across 589,000 square meters (11, 12). Curitiba was ranked at the time the ninth most populated city in Brazil, with estimated population of 1.8 million habitants, presenting the tenth highest Human Development Index (HDI) with 0.783, out of 5,565 municipalities of Brazil (13). Although categorized as a 100% urban area city, Curitiba has been environmentally friendly and frequently ranked as first in sustainability and quality of life in Brazil, with a high green-area ratio throughout more than 40 in-city parks and preservation areas (14). Curitiba has presented a classically humid subtropical highland climate (Köppen: Cfb), with mild winters (average 19°C) and summers (average 25°C) due to low latitude, apart from the typically temperate climate (15).

### 2.3 Blood samples

**Figure 1:**
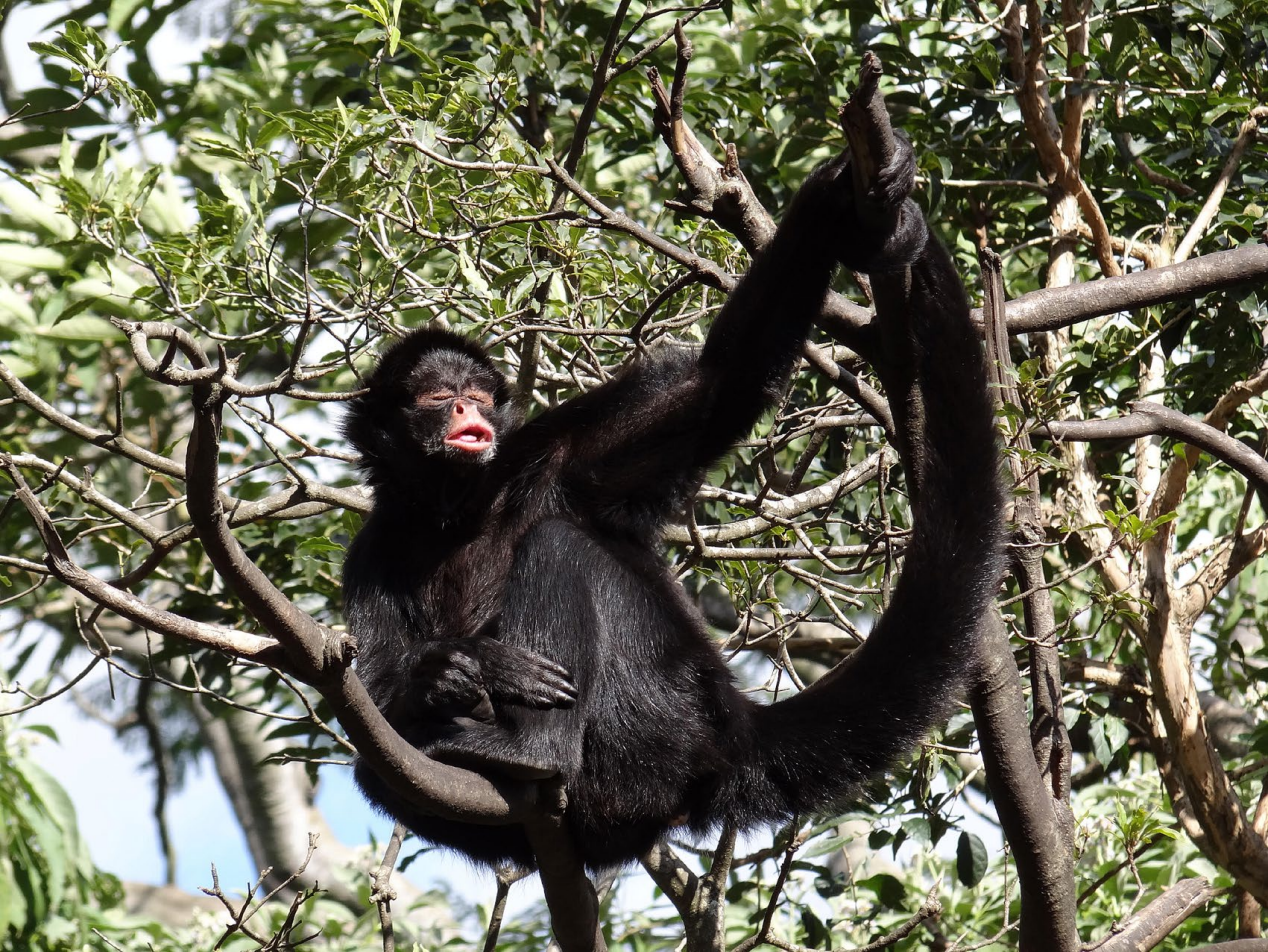
Captive black-faced black spider monkeys at the Zoological Garden of Curitiba, Paraná, Brazil.

**Figure 2:**
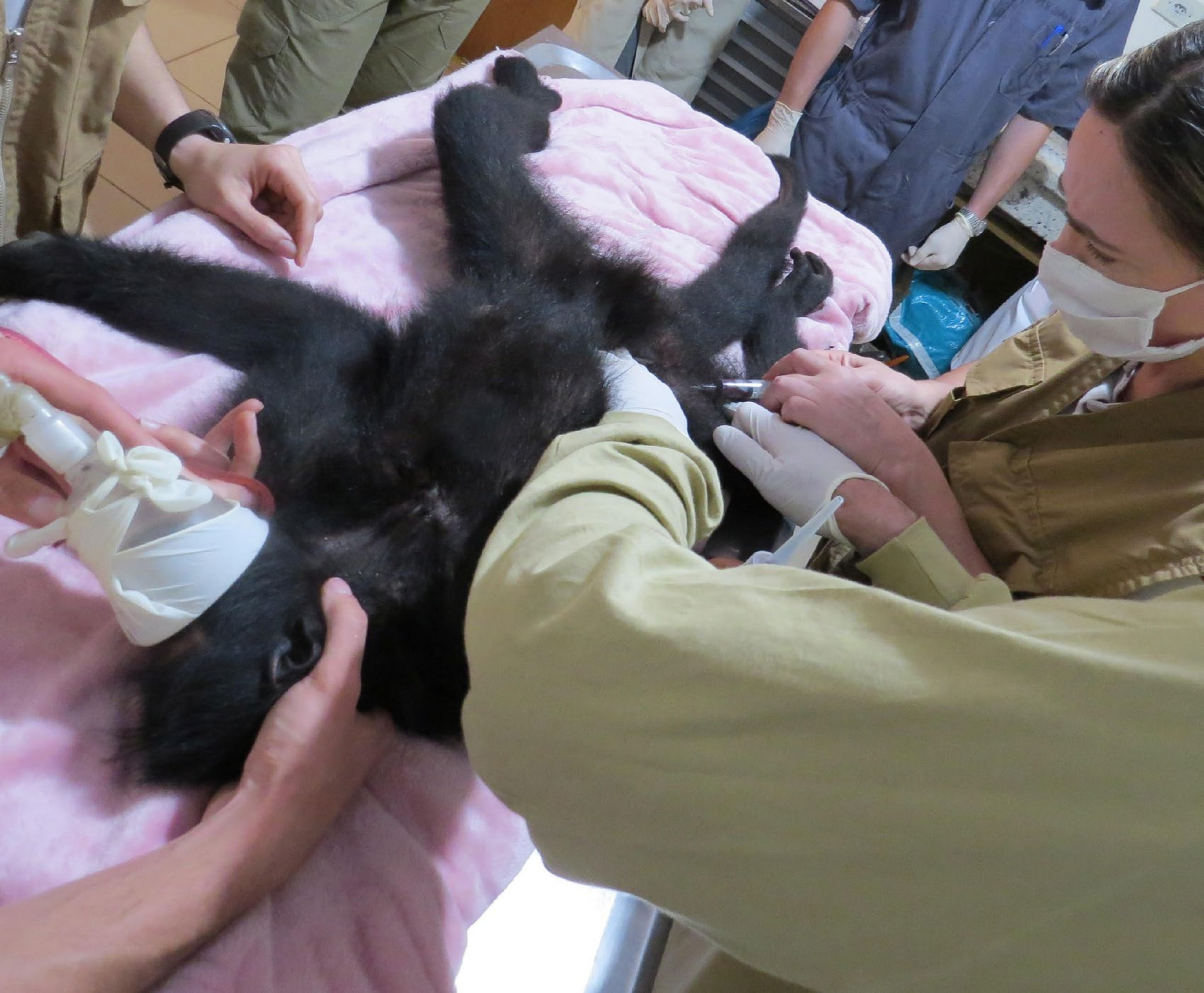
Blood samplings of black-faced black spider monkeys in the Veterinary Section, Zoological Garden of Curitiba, Paraná, Brazil.

Peripheral blood samples (3 mL) of black-faced black spider monkeys (Figure 1) were collected in 2021 by femoral puncture and performed by certified veterinarians, after physical restraint and inhalatory anesthesia with isoflurane (Figure 2). Blood samples were placed into EDTA tubes, separated into aliquots and stored at −20°C until testing.

### 2.4 Molecular analyses

DNA was extracted from blood using the commercial kit, DNeasy Blood & Tissue Kit (QIAGEN, Hilden, Germany), following manufacturer instructions.

A total of 5 μL of extracted DNA was submitted to Nested-PCR 18S rDNA as target in two rounds, with 8.5 uL of commercially available enzyme (Go Taq Master Mix Promega, Madison, WI, USA) used for the reaction, a premixed ready-to-use solution containing bacterially derived Taq DNA polymerase, dNTPs, MgCl2 and reaction buffers (16). A 0.5 μL volume of commercially available primers (Eurofins Genomics, Louisville, KY, USA) TRY R (5’CTACTGGGCAGCTTGGA3’) and F (5’GAAACAAGAAAC ACGGGAG3’) were used in the first round, and 0.5 μL of SSU R (5’CTGAGACTGTAACCTCAAAGC3’) and F (5’TGGGATAACAAA GGAGCA3’) in the second round. For the second round, 5 μL of amplified DNA with 1:10 of concentration was used. Both rounds were completed with 30 cycles, initial denaturation at 95°C for 3 min, followed by 30 cycles at 94°C for 30 s, 55°C for 60 s, 72°C for 90 s, 72°C for 10 min and kept at 4°C.

Amplified samples were purified using a commercial kit and Gel Band Purification (Illustra GFX PCR DNA GE Healthcare, Illinois, EUA), following the manufacturer protocol. Samples were sequenced by the Sanger method with a commercial kit (BigDye Terminator v3.1 Cycle Sequencing, Applied Biosystems, CA, USA) on a commercial sequencer (ABI 3730 DNA, Applied Biosystems, CA, USA) available at the PDTIS/Fiocruz sequencing platform. DNA sequences were analyzed using SeqManTM program 7.0 version (DNASTAR, Madison, Wisconsin, EUA) and BioEdit Sequence Alignment Editor v. 7.225 (17).

### 2.5 Phylogenetic analysis

The obtained consensus sequences were manually edited using the SeqMan-DNA Star Program and compared for similarity with sequences deposited in the GenBank database from the National Center for Biotechnology Information (NCBI), using the BLAST algorithm (Basic Local Alignment Search Tool). For species identification, the following values were adopted: cover (≥99%), identity (≥99%), and E-value (0.0). Sequence alignment was performed using the muscle method in the MEGA11 program, and the phylogenetic analysis of 18S rDNA gene sequences was performed by maximum likelihood (ML) to confirm the characterization of trypanosomatid species with the Kimura 2-parameter model and Gamma distribution to model evolutionary rate differences among sites [4 categories (+G, parameter = 0.2566)]. The percentage of trees in which the associated taxa clustered together was shown next to the branches of the figure.

The initial tree(s) for heuristic search were obtained automatically by applying Neighbor-Join and BioNJ algorithms to a matrix of pairwise distances estimated using the Maximum Composite Likelihood (MCL) approach, and then selecting the topology with superior log likelihood value. The tree was drawn to a scale, with branch lengths measured in the number of substitutions per site. Evolutionary analyses were conducted in MEGA11. To support the branch, ultrafast bootstrapping of 5,000 replications with 1,000 maximum interactions. Representative sequences from GenBank were used for the construction of the phylogenetic tree, among all the sequences obtained in this study.

## 3 Results

**Figure 3:**
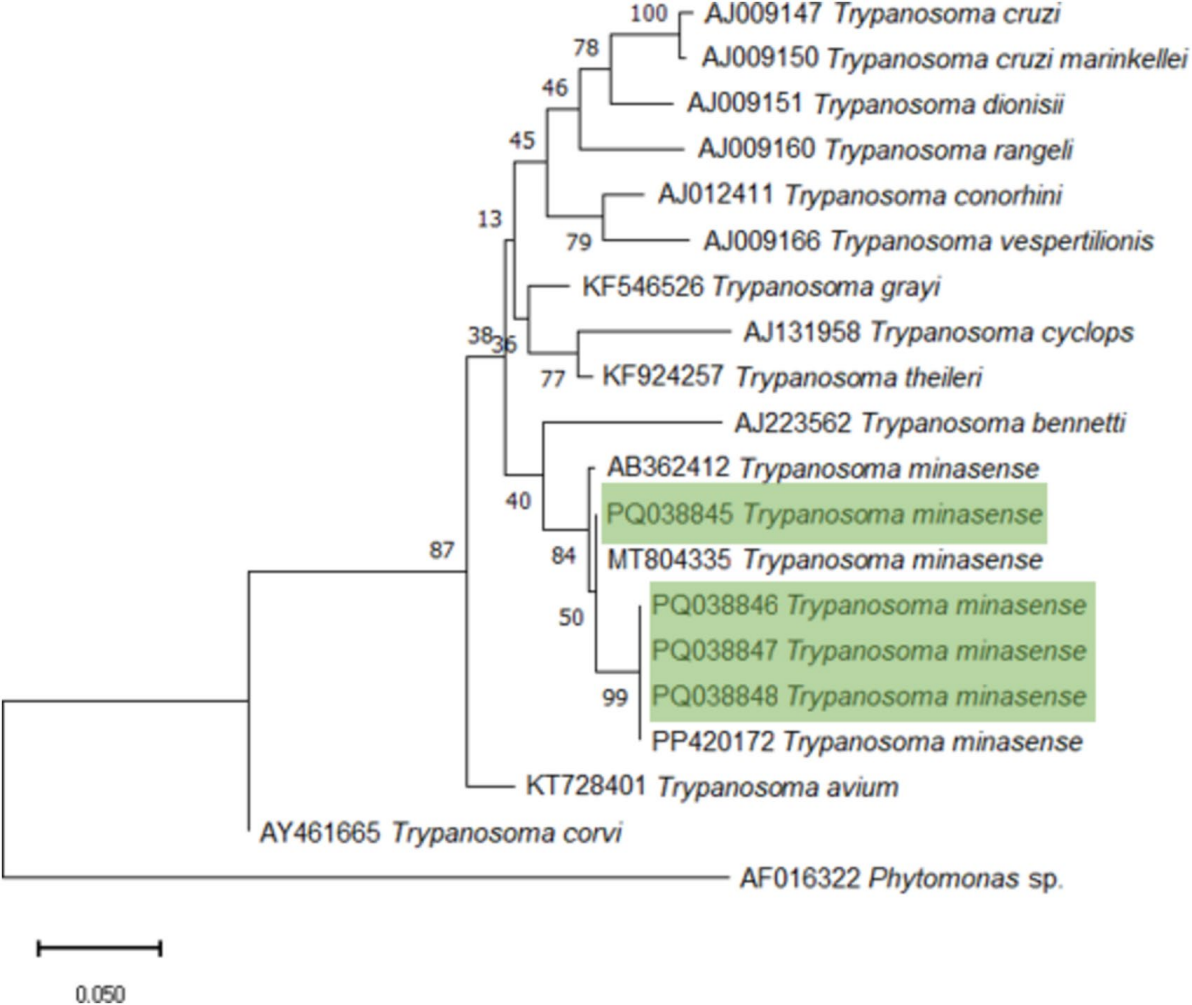
Phylogenetic tree of 18S rDNA gene sequences indicating the phylogenetic position of trypanosomatids characterized as *Trypanosoma minasense* (represented in green).

The full population of 17 healthy, black-faced spider monkeys kept at the time in the Zoological Garden of Curitiba were sampled, with 4/17 (23.5%) positive animals by PCR to *Trypanosoma* spp. infection, including two males and two females, with ages ranging from four to 25 years-old. All sampled animals were born at the Zoological Garden of Curitiba with no historic displacement. Although vertical transmission should be considered, both mothers of sampled animals have died long before samplings herein. Results of 18S rDNA sequencing have shown *Trypanosoma minasense* infection, confirmed by BLAST algorithm and deposited at GenBank (numbers PQ038845; PQ038846; PQ038847; PQ038848). A phylogenetic tree based on 18 S rRNA gene sequences was constructed (Figure 3). Mutations were considered synonymous, as the percentage of identity between the sequences obtained and the sequences deposited at NCBI presented nearly 100% identity (PQ038845 - Identity: 99.56%; PQ038846 - Identity: 99.64%; PQ038847 - Identity: 99.45%; PQ038848 - Identity: 99.11%).

## 4 Discussion

This is the first report of *T. minasense* in black-faced black spider monkeys worldwide and the first study accessing *Trypanosoma* species in NHP of southern Brazil.

In the present study, all positive primates were infected by *T. minasense*, trypanosomatid from Megatrypanum subgenera reported to infect several monkey families in South America (18). In Brazil, previous studies have shown natural infection by *T. minasense* in approximately 30 different Neotropical primates, including *Callithrix penicillata jordani* and *Callithrix geoffroyi* in southern (19), *Callithrix penicillata* trapped in southeastern (20), *Callithrix geoffroyi* (19), *Saimiri sciureus* in northern (21), *Sapajus paella* in central-western (9), and *Alouatta caraya* in southeastern Brazil (22). *T. minasense* was also previously detected in NHP of Brazilian botanical gardens and conservation institutions, including *Callithrix* spp. from Rio de Janeiro, southeastern region (23), and different species kept at the National Center of Primates, Pará State, northern Brazilian region (5). Infection of *T. minasense* was also reported in other South American countries, including *Alouatta caraya* in Argentina (24), *Alouatta palliata* in Costa Rica (25) and *Leontocebus weddelli* in Peru (26). Although very frequent in NHP from different geographical areas, the mode of transmission and potential vectors of *T. minasense* are still unknown (27).

*Trypanosoma minasense* has been classified as a primitive and non-pathogenic species, being commonly detected in NHP (28). As *T. minasense* may not be able to infect blood-sucking triatomines, information about which vectors could be involved or other transmission routes remains unclear (28). As primate hosts of *T. minasense* have been primarily arboreal, arboreous insects have been indicated as potential vectors (27). No clinical signs have been observed in naturally infected primates (29), nor human infection by *T. minasense* reported to date (20). All sampled animals herein were born in captivity at the Zoological Garden of Curitiba, excluding the possibility of previous or outside infection. Thus, the ecological environment in which the Zoological Garden of Curitiba is inserted, enable *T. minasense* transmission, either associated to an unknown vector or even derived from direct contact between these NHP. In addition, insects and other potential vectors should be further investigated by entomological surveillance and molecular testing, to fully establish *T. minasense* spreaders and reservoirs.

Herein, *Trypanosoma* spp. infection, including by *T. minasense*, was reported for the first time in spider monkeys. The black-faced black spider monkey has been currently classified as Endangered by the International Union for Conservation of Nature (30), mostly related to hunting and habitat loss due to agricultural expansion, with 50% decline in population over the past 45 years (30). In Brazil, this species has been distributed within transitional regions among Amazonia, Cerrado and Pantanal biomes (31). As previously suggested for different NHP species, the overall absence of clinical signs and low or null levels of pathogenicity have not itself supported additional risks and parasite impact on NHP species (23, 32), including the spider monkey population surveyed herein.

Although *T. minasense* has been classified as a primitive and non-pathogenic species commonly detected in NHP, the transmission routes and consequences for coinfection with *Trypanosoma cruzi* remain unclear. Thus, surveillance of *Trypanosoma* spp. infection should be always considered in both captive and free-range non-human primates to establish such transmission routes, besides host and vector species involved.

## Data Availability

The datasets generated for this study can be accessed in GenBank nucleotide database (NCBI repository) under the following numbers; PQ038845, PQ038846, PQ038847 and PQ038848.
